# Characterization of leukotrienes in a pilot study of older asthma subjects

**DOI:** 10.1186/1742-4933-7-8

**Published:** 2010-07-05

**Authors:** Sharmilee M Nyenhuis, Elizabeth A Schwantes, Sameer K Mathur

**Affiliations:** 1 Department of Medicine, Section of Allergy, Pulmonary and Critical Care, University of Wisconsin, Madison, WI, USA; 2 Department of Medicine, Section of Pulmonary, Critical Care, Sleep and Allergy, University of Illinois-Chicago, Chicago, IL, USA

## Introduction

Asthma is a chronic inflammatory disorder of the lower airway that results in mucus secretion, airway edema and reversible airway obstruction [1]. These characteristic features lead to the clinical symptoms of asthma, which include recurrent episodes of wheezing, breathlessness, chest tightness and coughing [2]. The pathophysiology of asthma is not fully elucidated but the current paradigm involves multiple cell types (ie. eosinophils, neutrophils, mast cells, T-cells) and their mediators.

The most recent estimates of asthma prevalence in those over the age of 65 are between 4 and 8%, which may be an underestimate due to an underdiagnosis of asthma in the elderly [3]. An increase in morbidity and mortality and a reduced response to bronchodilators in the emergency department setting have been shown in older asthmatics compared to their younger counterparts [4-7]. Elderly asthmatics also have a higher rate of severe exacerbations, emergency department visits, and hospitalizations than younger asthmatics [8,9].

Leukotrienes are potent pro-inflammatory lipid mediators that have been shown to have a role in asthma [10]. Leukotrienes are a product of arachidonic acid via the 5-lipoxygenase pathway. Leukotriene B_4 _(LTB_4_) is a potent chemotactic agent and activator of neutrophils and is also produced mainly by neutrophils. The presence of LTB_4 _in the lung results in neutrophil recruitment and activation leading to superoxide anion generation and cholinergic airway hyperresponsiveness [11]. The cysteinyl leukotrienes (CysLTs), LTC_4_, LTD_4_, and LTE_4_, are known for their profound effect on the airway, including increased microvascular permeability and vasodilation, airway smooth muscle contraction, mucous secretion and impaired mucociliary clearance [10]. CysLTs are produced by eosinophils, basophils, mast cells, macrophages and to a lesser degree by T cells and endothelial cells [10,12]. Increased CysLT levels have been detected in the sputum of patients with asthma and have been shown to correlate with symptom severity [13].

Despite older asthmatics often having more severe disease and exacerbations, few studies have been done to characterize their asthma at a cell and/or molecular level. We have previously shown that functional differences exist in eosinophils from older adult asthmatics *in vitro*; specifically, diminished IL-5 stimulated eosinophil derived neurotoxin (EDN) release and superoxide production [14]. In this pilot study, we sought to compare pro-inflammatory lipid mediator production, specifically leukotrienes LTB_4 _and CysLT, both *in vitro *and *in vivo *in young and older adult asthmatics. Identifying the inflammatory differences seen in older asthmatics may be important for the diagnosis, improving the morbidity and mortality, as well as determining optimal therapies of the disease in this growing population.

## Methods

We recruited subjects in two age groups, 20 to 40 years (n = 12) and 50 to 70 years (n = 6), in a study protocol approved by the University of Wisconsin Health Sciences Institutional Review Board. Inclusion criteria included a physician diagnosis of mild to moderate asthma with a provocative concentration of methacholine causing a 20% fall in FEV_1 _(PC_20_) of < 8 mg/mL or albuterol reversibility on spirometry of ≥ 12%. Exclusion criteria included history of tobacco use > 5 pack-years or use in the previous year, prednisone use within 1 month, donation of blood (greater than 1/2 pint) in the previous 8 weeks, FEV_1 _< 60%, severe asthma, upper and/or lower respiratory tract infection in the previous 4 weeks, diabetes, pregnancy, or an active cardiovascular disease other than controlled hypertension.

At the study visit, medical history, spirometry with bronchodilator reversibility, sputum induction, allergy skin prick test, physician exam and phlebotomy were performed. Sputum processing was performed as described previously [15]. LTB_4_, LTC_4 _and CysLT levels in culture supernates or the sputum samples were measured using the LTB_4_, LTC_4 _and CysLT competitive EIA kits, respectively (Cayman Chemical, Ann Arbor, MI). Heparinized venous blood was lysed in a hypotonic solution and subjected to gradient centrifugation. Neutrophils were isolated from the granulocyte pellet and were > 95% pure and > 98% viable. Eosinophils were also isolated from the granulocyte pellet by negative selection using anti-CD16, anti-CD14, and anti-CD3 magnetic beads (Miltenyi Biotechnology; Auburn,CA) as described previously [16]. The eosinophils were typically > 99% pure and > 98% viable.

## Results

**Figure 1:**
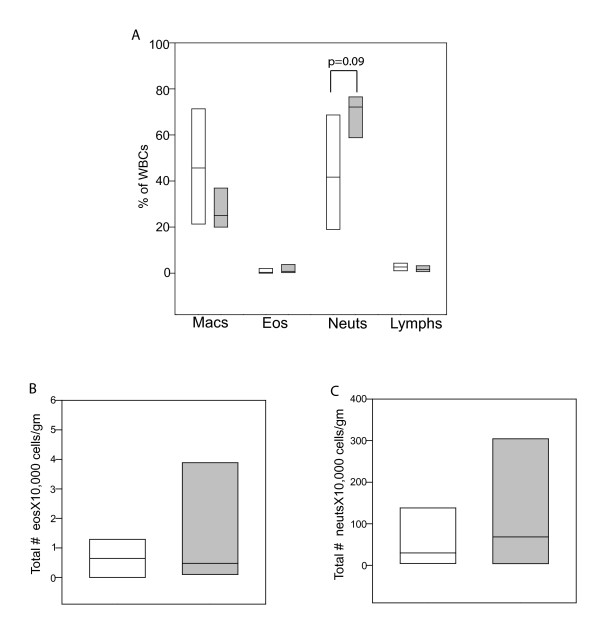
**Sputum cell type differentials**. Box plots of sputum cell percentages (**1A**) and total eosinophil (**1B**) and neutrophil (**1C**) number are shown for young subjects (white bars; n = 11), and older subjects (gray bars; n = 6). One young subject had > 80% epithelial cells in the sputum (indicating oral/salivary contamination) and was not included in the sputum differential. Comparisons between the young and older groups were performed with *t *test analysis or Mann-Whitney rank sum analysis. Statistical significance was defined at p < 0.05. Mac = macrophage; Eos = eosinophil; Neut = neutrophil; Lymph = lymphocyte; Median: ----- (solid)

Table 1 shows a comparison of lung function and peripheral blood characteristics for the subjects in the study. Both FEV_1 _and FVC were significantly lower in the older group compared to the young subjects, which is consistent with lung volume decreases in the aging population [17]. However, percentage of predicted values, for both FEV_1 _and FVC were comparable between young and older groups, suggesting similar disease severity in both age groups. There was no difference in peripheral blood eosinophil counts between young and older asthmatics. Sputum cell differentials revealed a tendency for increased percentage of neutrophils in older asthma subjects, 72(65-76)%, compared to young asthma subjects, 42(21-66)%, p = 0.09 (Figure 1A). This is consistent with our previous cohort of older asthmatics exhibiting a significantly higher percentage of sputum neutrophils [18]. The total number of eosinophils in the sputum were similar in both the young and older group [Young: 0.6 (0-1.3) × 10,000 cells/gm; Older: 0.5 (0.1-3.9) × 10,000 cells/gm; Figure 1B], while the total numbers of neutrophils apparently increased though not statistically significant [Young: 30 (4-138) × 10,000 cells/gm; Older 68 (4-304) × 10,000 cells/gm; Figure 1C].

**Table 1:** Subject Characteristics

	Young (n = 12)	Older (n = 6)
Age (years, range given)*	27 (20-35)	64 (58-70)

Duration of disease	17 (6-30)	37 (4-65)

FEV_1 _(L)*	3.35 (+/- 0.81)	2.24(+/- 0.28)

FEV_1 _% Predicted	86 (+/- 14.7)	81 (+/- 16.46)

FVC (L)*	4.28 (3.99-5.02)	3.24 (+/- 0.71)

FVC % Predicted	100 (+/- 12.15)	89 (+/- 12.62)

WBC (10^6^cells/mL)	5.55 (4.75-6.85)	5.6 (5.13-6)

Absolute Eosinophil Count (cells/mL)	204 (+/- 127.75)	114 (+/- 82.95)

Positive Allergy Testing^+^	11/12	6/6

Inhaled Corticosteroid Use	6/12	4/6

Data are presented as mean values (+/- standard deviation; t-test) or median values (25%-75% interval; Mann-Whitney Rank-Sum). *Statistically significant difference between young subjects and older subjects (p < 0.05). ^+^At least one positive skin prick test result for grass, mold, ragweed, tree, cat, dog, or dust mite.

**Figure 2:**
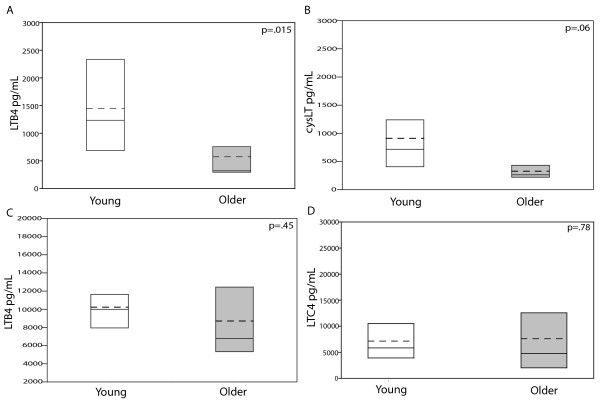
***In vivo and in vitro *leukotriene levels**. Comparisons between the young and older groups were performed with *t *test analysis or Mann-Whitney rank sum analysis. Statistical significance was defined at p < 0.05. White bars: young subjects; Gray bars: older subjects Mean: - - -(dashed) Median: ----- (solid). **2A and 2B**. Sputum was processed and LTB_4 _and CysLT levels were measured using the LTB_4 _and CysLT competitive EIA kit, respectively (Cayman Chemical, Ann Arbor, MI). Sputolysin (Calbiochem) was added to all assay standards to represent an equivalent amount in the diluted sputum samples and run in duplicate. Standard curves for LTB_4 _and CysLT were performed both with and without Sputolysin to ensure an equivalent slope was obtained. Young: n = 11 (One young subject had > 80% epithelial cells in the sputum and was not included in the biochemical analysis.); Older: n = 6. **2C and 2D**. Peripheral blood neutrophils and eosinophils were isolated and stimulated with calcium ionophore (A23187) for 60 min at 37°C. LTB_4 _and LTC_4 _were measured by competitive EIA kit (Cayman Chemical, Ann Arbor, MI). Young: n = 12; Older: n = 6

*In vivo *levels of LTB_4 _and CysLT were measured in the induced sputum samples from young and older asthma subjects. Sputum LTB_4 _levels in older asthma subjects, 320 (301-397) pg/mL, were significantly decreased compared to the young subjects, 1235 (706-2259) pg/mL, p = 0.015 (Figure 2A). Sputum CysLT levels in older asthma subjects, 264 (224-269) pg/mL, were lower than in younger asthma subjects, 717 (408-1152) pg/mL; however, this only approached statistical significance, p = 0.06 (Figure 2B).

In order to determine whether these *in vivo *differences were due to an age-related diminished ability of neutrophils or eosinophils to produce leukotrienes, both purified neutrophils and eosinophils were treated with calcium ionophore to stimulate leukotriene production. As shown in Figure 2C, the neutrophil production of LTB_4 _from older asthma subjects, 8708 ± 5326 pg/mL, was comparable to the young subjects, 10234 ± 3078 pg/mL, p = 0.45. Eosinophil production of LTC_4 _from young, 5836 (3678-9512) pg/mL, and older asthma subjects, 4766 (2685-8456) pg/mL, was also similar in the two groups, p = 0.78 (Figure 2D).

**Figure 3:**
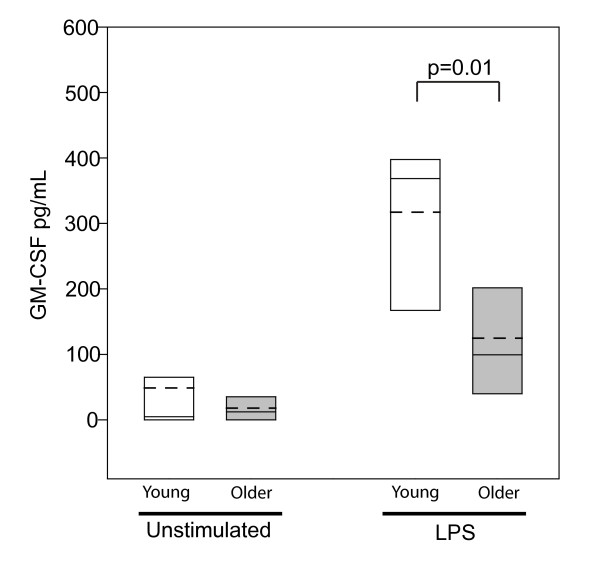
**Production of GM-CSF in PBMCs; Peripheral blood mononuclear cells (PBMCs) were either not stimulated or stimulated with LPS (0.4 μg/mL) and supernatants analyzed by ELISA for GM-CSF**. Comparisons between the young and older groups were performed with *t *test analysis. Statistical significance was defined at p < 0.05. White bars: young subjects (n = 12); Gray bars: older subjects (n = 6); Mean: - - - (dashed) Median: ----- (solid)

Since GM-CSF can stimulate leukotriene production by neutrophils and eosinophils[11] and changes in GM-CSF mediated signaling have been shown to occur in elderly human subjects *in vitro*[19,20], we examined GM-CSF production by peripheral blood mononuclear cells (PBMCs) in the older and young asthma subjects. As shown in Figure 3, GM-CSF production by unstimulated PBMC was similar; however, LPS-stimulated PBMCs from the older asthma subjects, 125 ± 118 pg/mL, produced significantly less GM-CSF than their younger counterparts, 317 ± 136 pg/mL, p = 0.01.

## Discussion

To our knowledge, a characterization of leukotriene levels has not been previously performed in an older adult asthma population. In this pilot study of young and older mild-to-moderate asthmatics we found that older asthma subjects had lower *in vivo *levels of LTB_4 _and CysLT in the sputum at baseline disease. This difference in leukotriene levels was not a reflection of fewer eosinophils and neutrophils in the airway as the total number of these cells in the sputum were similar or greater in our older asthmatic group. However, young and older asthma subjects produced comparable amounts of LTB_4 _and LTC_4 _*in vitro *when neutrophils and eosinophils, respectively, were stimulated with calcium ionophore. The observation that LPS stimulation of PBMCs resulted in less GM-CSF production in the older asthma subjects provides a potential explanation for the age-related differences in sputum leukotriene levels. Rather than an intrinsic defect in neutrophils and eosinophils, there may be less GM-CSF in the airways of older asthmatics serving as a stimulant to produce leukotrienes. However, it is possible that other age-related changes in the *in vivo *inflammatory milieu contribute to the diminished levels of leukotrienes in the airway.

There are several limitations to this study including the absence of a control non-asthmatic population, lack of subjects >65 years old, and few total number of subjects enrolled. Though multiple studies have consistently revealed increases in leukotriene production in asthmatic airways compared to controls, we cannot fully gauge the magnitude of our findings in the older asthmatic without non-asthmatic older controls [21]. It is possible though that our findings would have been more profound if we had more subjects in an even older (> 65 years old) population. Furthermore, as a pilot study, we had a limited number of subjects enrolled in order to establish preliminary observations to serve as the focus of future studies.

Calcium ionophore is a potent activator of leukotriene production in eosinophils and neutrophils. It is possible that we were unable to detect small differences in leukotriene production *in vitro *with the use of such a potent stimulator of leukotriene production. Therefore, a less potent, physiologically-relevant stimulant (such as GM-CSF) could reveal a difference in leukotriene production *in vitro*.

The use of inhaled glucocorticoids by some subjects in our study may represent a confounder as glucocorticoids can have multiple effects such as a decrease in inflammatory mediators and neutrophil apoptosis [22]. However, glucocorticoids have not been shown to affect LTB_4 _formation *in vitro *and *in vivo *[23]. Also, we performed a statistical analysis of paired young and older subjects that were matched based on inhaled corticosteroid use, which continued to show statistically significant differences in the *in vivo *leukotriene levels (data not shown). Thus, the use of inhaled glucocorticoids alone cannot explain our findings.

The lower levels of CysLTs in the airways of older adults may have an impact on the effectiveness of CysLT receptor antagonists in the older population. Two studies to date have examined the efficacy of the CysLT receptor antagonists in an older adult population and concluded that their effectiveness might be limited or altered in older asthmatics [24,25]. Furthermore, the neutrophil predominance found in the airway of older asthmatics may actually represent a tendency for decreased responsiveness to glucocorticoids as has been observed in the neutrophilic phenotype of severe asthma [26].

Our findings show that aging can result in changes in the airway environment in asthmatics, specifically an increase in airway neutrophils and decreases in both LTB_4 _and CysLT levels at baseline. This characterization of leukotrienes in older adult asthmatics reveals significant differences that may have clinical relevance not only in baseline asthma but also during an exacerbation of disease. Understanding the biological changes of airway inflammation in the aging population will aid in the development of future therapies and impact the increased morbidity and mortality that is associated with this phenotype of asthma.

## Abbreviations

CysLTs: cysteinyl leukotrienes; LTB_4_: leukotriene B_4_; PBMCs: peripheral blood mononuclear cells; GM-CSF: granulocyte macrophage colony-stimulating factor; EDN: eosinophil derived neurotoxin; FVC: forced vital capacity; FEV_1_: forced expiratory volume in 1 second; LPS: lipopolysaccharide

## Competing interests

SMN received a fellowship award co-sponsored by GlaxoSmithKline, which helped fund this study.

## Authors' contributions

SMN performed the leukotriene and GM-CSF immune assays, analyzed data, created graphs and drafted the manuscript. EAS performed cell separations, sputum processing, sputum differentials, assisted with immune assays, and assisted with data analysis and drafting of the manuscript. SKM conceived of the study, and participated in its design and coordination and helped to draft the manuscript. All authors reviewed and approved the final manuscript.
